# Supplementary material for: Normal male fertility in a mouse model of KPNA2 deficiency
Source: PLoS One. 2024 Oct 18;19(10):e0304189. doi: 10.1371/journal.pone.0304189 (PMC11488728; doi:10.1371/journal.pone.0304189)
Supplement: S2 File — (PDF) [file pone.0304189.s002.pdf]

For Fig 1B

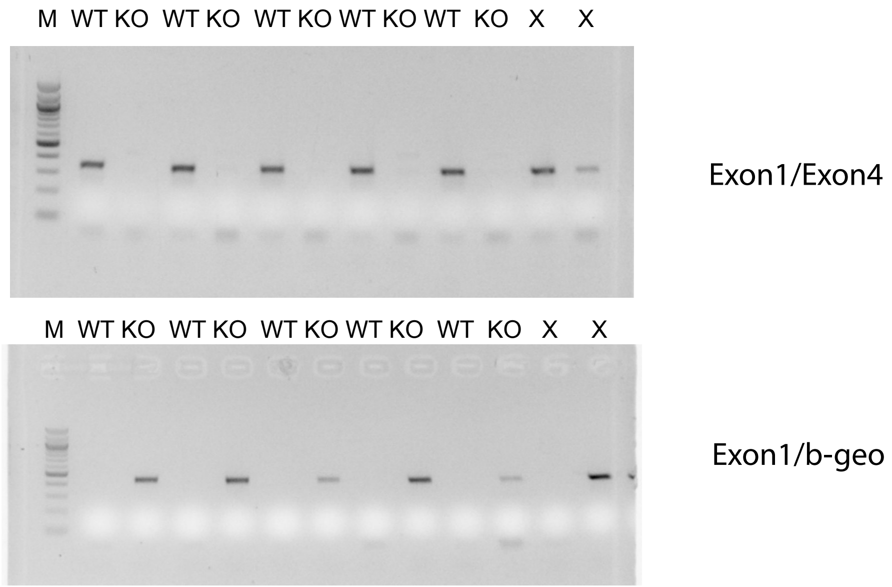

For Fig 1C

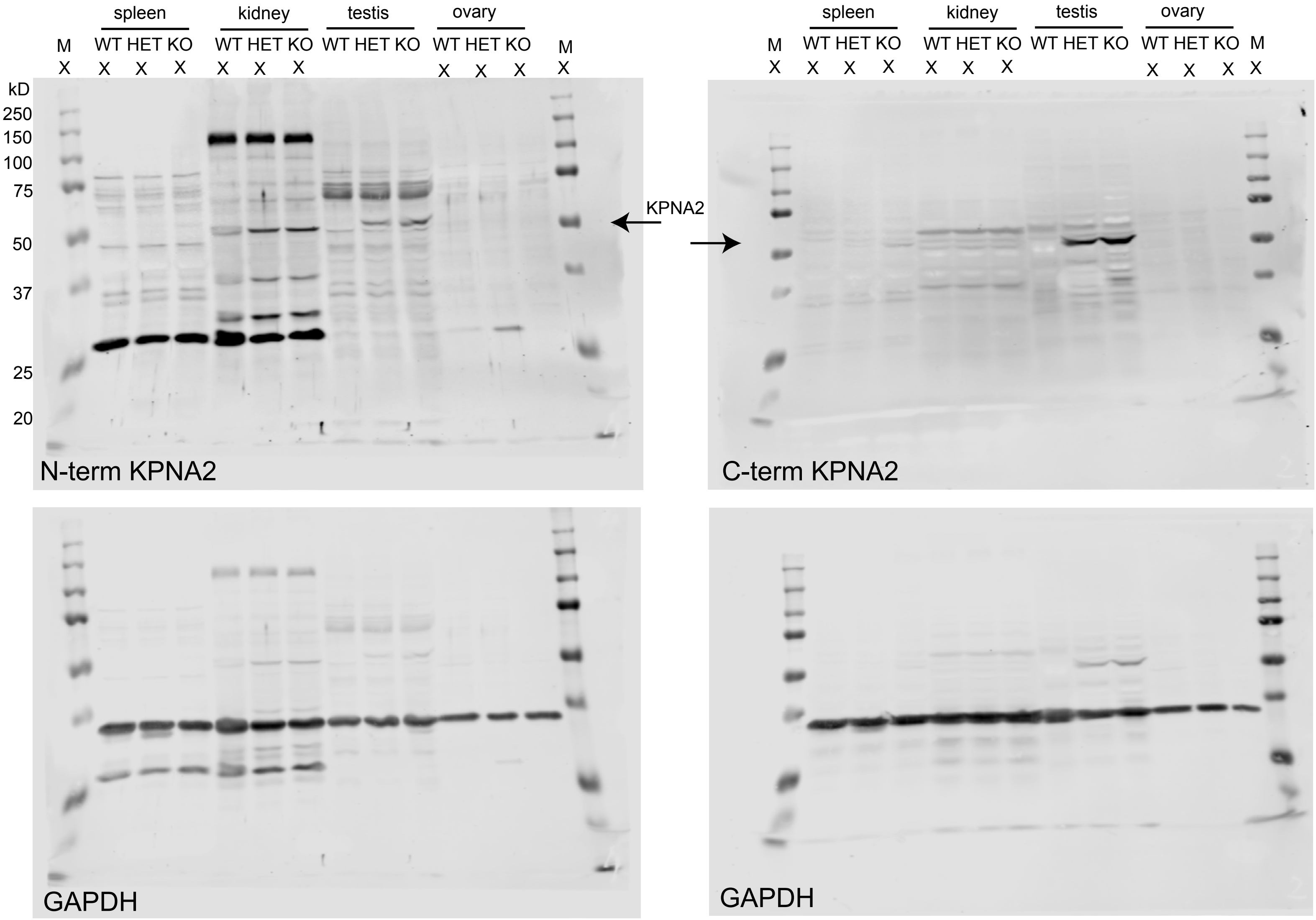

for the Figure, only lanes with testis extracts were shown

Marker: Precision Plus Protein All Blue standards (Bio-Rad)
