# Supplementary material for: Normal male fertility in a mouse model of KPNA2 deficiency
Source: PLoS One. 2024 Oct 18;19(10):e0304189. doi: 10.1371/journal.pone.0304189 (PMC11488728; doi:10.1371/journal.pone.0304189)
Supplement: Attachment — Submitted filename: Response to reviewers.docx [file pone.0304189.s003.docx]

Dear academic editor, dear reviewers,

We wish to thank you for the valuable feedback and the time you have dedicated to improve the manuscript.

Journal Requirements:

When submitting your revision, we need you to address these additional requirements.

1. Please ensure that your manuscript meets PLOS ONE's style requirements, including those for file naming. The PLOS ONE style templates can be found at 
https://journals.plos.org/plosone/s/file?id=wjVg/PLOSOne_formatting_sample_main_body.pdf and 
<https://journals.plos.org/plosone/s/file?id=ba62/PLOSOne_formatting_sample_title_authors_affiliations.pdf>

We have adjusted the manuscript according to the requirements

2. To comply with PLOS ONE submissions requirements, in your Methods section, please provide additional information regarding the experiments involving animals and ensure you have included details on (1) methods of sacrifice, and (2) efforts to alleviate suffering.

The informations have been added.

3. We note that your Data Availability Statement is currently as follows: [All relevant data are within the manuscript and its Supporting Information files.]

Please confirm at this time whether or not your submission contains all raw data required to replicate the results of your study. Authors must share the “minimal data set” for their submission. PLOS defines the minimal data set to consist of the data required to replicate all study findings reported in the article, as well as related metadata and methods (https://journals.plos.org/plosone/s/data-availability#loc-minimal-data-set-definition).

For example, authors should submit the following data:

- The values behind the means, standard deviations and other measures reported;
- The values used to build graphs;
- The points extracted from images for analysis.

We have included a supplementary file with original data.

Authors do not need to submit their entire data set if only a portion of the data was used in the reported study. If your submission does not contain these data, please either upload them as Supporting Information files or deposit them to a stable, public repository and provide us with the relevant URLs, DOIs, or accession numbers. For a list of recommended repositories, please see https://journals.plos.org/plosone/s/recommended-repositories.

If there are ethical or legal restrictions on sharing a de-identified data set, please explain them in detail (e.g., data contain potentially sensitive information, data are owned by a third-party organization, etc.) and who has imposed them (e.g., an ethics committee). Please also provide contact information for a data access committee, ethics committee, or other institutional body to which data requests may be sent. If data are owned by a third party, please indicate how others may request data access.

We have included a supplementary file with original data.

4. PLOS ONE now requires that authors provide the original uncropped and unadjusted images underlying all blot or gel results reported in a submission’s figures or Supporting Information files. This policy and the journal’s other requirements for blot/gel reporting and figure preparation are described in detail at https://journals.plos.org/plosone/s/figures#loc-blot-and-gel-reporting-requirements and https://journals.plos.org/plosone/s/figures#loc-preparing-figures-from-image-files. When you submit your revised manuscript, please ensure that your figures adhere fully to these guidelines and provide the original underlying images for all blot or gel data reported in your submission. See the following link for instructions on providing the original image data: <https://journals.plos.org/plosone/s/figures#loc-original-images-for-blots-and-gels>.

A supplementary file with orginal blots and gels was added (S2)

In your cover letter, please note whether your blot/gel image data are in Supporting Information or posted at a public data repository, provide the repository URL if relevant, and provide specific details as to which raw blot/gel images, if any, are not available. Email us at plosone@plos.org if you have any questions.

A supplementary file with orginal blots and gels was added (S2)

5. Please include your full ethics statement in the ‘Methods’ section of your manuscript file.

or not you obtained informed written or verbal consent. If consent was waived for your study, please include this information in your statement as well.

An ethics statement was included in the M&M section. For the animal experiments performed in this study, an approval by the ethics commetee has not been necessary at this time. The sacrifice of the animals was reported under §4 of the German animal wellfare act (TierSchG), which has been reformed meanwhile.

6. Please review your reference list to ensure that it is complete and correct. If you have cited papers that have been retracted, please include the rationale for doing so in the manuscript text, or remove these references and replace them with relevant current references. Any changes to the reference list should be mentioned in the rebuttal letter that accompanies your revised manuscript. If you need to cite a retracted article, indicate the article’s retracted status in the References list and also include a citation and full reference for the retraction notice.

The reference was changed to the peer review one (Ref # 23)

Reviewers' comments:

Reviewer's Responses to Questions

**Comments to the Author**

1. Is the manuscript technically sound, and do the data support the conclusions?

The manuscript must describe a technically sound piece of scientific research with data that supports the conclusions. Experiments must have been conducted rigorously, with appropriate controls, replication, and sample sizes. The conclusions must be drawn appropriately based on the data presented.

Reviewer #1: Yes

Reviewer #2: Yes

2. Has the statistical analysis been performed appropriately and rigorously?

Reviewer #1: Yes

Reviewer #2: Yes

3. Have the authors made all data underlying the findings in their manuscript fully available?

The [PLOS Data policy](http://c05y1x9s.r.us-east-2.awstrack.me/L0/http:%2F%2Fwww.plosone.org%2Fstatic%2Fpolicies.action%23sharing/1/010f019137ad4d54-8bb452e3-5c62-47ce-af5e-b3bbd34291c1-000000/J9tvrpwwFujUpxluB0vUWen12rg=171) requires authors to make all data underlying the findings described in their manuscript fully available without restriction, with rare exception (please refer to the Data Availability Statement in the manuscript PDF file). The data should be provided as part of the manuscript or its supporting information, or deposited to a public repository. For example, in addition to summary statistics, the data points behind means, medians and variance measures should be available. If there are restrictions on publicly sharing data—e.g. participant privacy or use of data from a third party—those must be specified.

Reviewer #1: No

Reviewer #2: Yes

4. Is the manuscript presented in an intelligible fashion and written in standard English?

PLOS ONE does not copyedit accepted manuscripts, so the language in submitted articles must be clear, correct, and unambiguous. Any typographical or grammatical errors should be corrected at revision, so please note any specific errors here.

Reviewer #1: Yes

Reviewer #2: Yes

5. Review Comments to the Author

Please use the space provided to explain your answers to the questions above. You may also include additional comments for the author, including concerns about dual publication, research ethics, or publication ethics. (Please upload your review as an attachment if it exceeds 20,000 characters)

**Reviewer #1:**

In the present manuscript, the authors were interested in deciphering the role of one of the karyopherin alpha family proteins, KPNA2, implicated in protein nuclear transport, in male germ cell development and fertility in mice. Authors have previously shown that KPNA2 is essential for early embryonic development in mice, which deficiency results in female infertility. The present work and the previous one were based in a genetically modified mouse model, KPNA2 KO, produced by gene-trapping in embryonic stem cells. The main conclusion is that KPNA2 is dispensable for spermiogenesis, and its absence does not affect male fertility. However, some changes were observed comparing to WT littermate males: sperm counting was significantly reduced, head/neck sperm abnormalities were seen, and males showed a reduced body weight.

REVIEWER COMMENTS:

Below, I have some suggestions and comments with the aim to make the M&M section more complete and the Results section clearer. I hope the authors can take them into consideration. Manuscript should have had number lines to make correction easier.

We have added line numbers to the manuscript.

MATERIALS AND METHODS

Generation of KPNA2 KO mice. I suggest the authors should refer the generation of this KO line to the work in which it was first published (Rother, F. et al, 2024. Karyopherin α2 is a maternal effect gene required for early embryonic development and female fertility in mice. FASEB Journal. 2024;38:e23623) because this model was already used by them in a previous work.

We have referred to the suggested FASEB publication.

Although Animal Ethics statement was uploaded on the submission process, I would suggest to add this information to the M&M section.

An Ethics statement was included in M&M.

Regarding blastocyst injection with ESC, there are some missing details. I suggest the authors could add the strain of the ESC clone purchased at MMRRC and details of the following procedures: superovulation of blastocyst donor females (hormones, times), blastocyst collection and embryo transfer (surgical or non-surgical technique was used? Reagents and doses of anesthesia and analgesia if used). Please detail the mouse strain/substrain of the blastocyst donor females and recipient females.

All information have been added to the M&M.

The name of the strain C57BL/6 is misspelled, correct the “L”. In the Discussion section, it appears that the C57BL/6N strain was used, please use the name of this substrain in the M&M section.

The typo was corrected.

Details regarding body weight analysis are completely missing. How many animals of each age and genotype were evaluated?

The information was included in the text. Additionally, the raw data are provided in S1 file that show how many animals were used.

The procedures regarding breeding of KPNA2 KO mice to evaluate fertility should be added. How many matings were tested, type of mating (1 female x 1 male; 2 females x 1 male; etc), duration of the mating (weeks, months).

The information was included in the M&M section.

Following PLOS One policy, all data underlying the findings in the manuscript should be fully available. I suggest the authors to report all the data in tables as supplemental files: Body weight of male mice, litter size, epididymal sperm count and sperm morphology.

A raw data table was attached as supplemental file (S1).

RESULTS

For a clearer understanding of the results, the mean numbers of the following parameters, for each tested group, would be added to the main text: litter size; epididymal sperm counts; normal and head/neck abnormal sperm percentages. It complements the information showed in the graphs.

We have added the mean numbers in the text.

DISCUSSION

Please, avoid the term “fathers” by explaining the different matings evaluated.

The term was substituted by” males”

I suggest changing the following phrase: “Differently observed phenotypes in KO mice” to “Different phenotypes in KO mice…”

We made the changes according to the suggestion.

Please, correct the B6 strain name (C57BL/6).

The typo was corrected.

The following phrase should be corrected to be understandable: This leaves around 14% of sperms (compared to WT) with a normal morphology, which is likely to be sufficient for fertilizing single oocytes, as it proved to be the case in our mouse model. Where does the 14% came out?

A clearer sentence was inserted.

In Wang et al. 2023 is concluded that KPNA2 in mice could play the role of KPNA7 in humans, which deficiency has been related to preimplantation embryo arrest. Deficiency of KPNA7 in male humans, has been studied/reported? If so, a brief comment could be added to the discussion section.

To our knowledge, no studies/data exist about KPNA7 deficiency or mutations in male humans.

FIGURE LEGENDES
Correct “und” by “and” in Figure B) RT-PCR of KPNA2

Corrections were made

REFERENCE LIST
I suggest changing reference #23 to the peer-reviewed one (The FASEB Journal. 2024;38:e23623).

The reference has been changed to the peer-reviewed one.

Reviewer #2: The MS submitted by Rother et al. describes in an elegant way the characterization of a KPNA2 KO mice regarding the fertility in males. This protein has been already studied in female mice leading to a deffect in embryo development, and in KO male mice leading to infertlity, in this case generated by another research group. In the present MS the males did not show signs of unfertility, although sperm count and abnormalities were similar to the previous reported model. The main difference can be attributed to the genetic background of the mice, something that accounts for multiple difference among mice strain and subtrains. Language is very clear and the experiments have been carried out appropiately with a proper discussion.

In my opinion, the MS can be published in its present form.

6. PLOS authors have the option to publish the peer review history of their article ([what does this mean?](https://c05y1x9s.r.us-east-2.awstrack.me/L0/https:%2F%2Fjournals.plos.org%2Fplosone%2Fs%2Feditorial-and-peer-review-process%23loc-peer-review-history/1/010f019137ad4d54-8bb452e3-5c62-47ce-af5e-b3bbd34291c1-000000/cHa3efKdrzKVsLDitRSuYnqAhWY=171)). If published, this will include your full peer review and any attached files.


If you choose “no”, your identity will remain anonymous but your review may still be made public.


**Do you want your identity to be public for this peer review?** For information about this choice, including consent withdrawal, please see our [Privacy Policy](https://c05y1x9s.r.us-east-2.awstrack.me/L0/https:%2F%2Fwww.plos.org%2Fprivacy-policy/1/010f019137ad4d54-8bb452e3-5c62-47ce-af5e-b3bbd34291c1-000000/LVPlbYJR_AJso-wRKmGKDHvrC1o=171).

Reviewer #1: No

Reviewer #2: **Yes:** Martina Crispo
